# Mosaic *TP53* pathogenic variant in early-onset breast cancer: a case report

**DOI:** 10.3389/fonc.2026.1814371

**Published:** 2026-05-08

**Authors:** Luana Greco, Chiara Miggiano, Arianna Dal Buono, Monica Barile, Riccardo Borroni, Giovambattista Rodà, Armando Santoro, Alessandro Repici, Pietro Cavalli, Paolo Bianchi

**Affiliations:** 1 Operational Unit (O.U.) Medical Genetics Laboratory, Oncological Molecular Genetics section, Istituto di Ricovero e Cura a Carattere Scientifico (IRCCS) Humanitas Research Hospital, Rozzano, Milan, Italy; 2 Humanitas Cancer Center, Istituto di Ricovero e Cura a Carattere Scientifico (IRCCS) Humanitas Research Hospital, Rozzano, MI, Italy; 3 Department of Biomedical Sciences, Humanitas University, Pieve Emanuele, MI, Italy; 4 Department of Gastroenterology, Istituto di Ricovero e Cura a Carattere Scientifico (IRCCS) Humanitas Research Hospital, Rozzano, Milan, Italy; 5 Dermatology Unit, Humanitas Research Hospital-Istituto di Ricovero e Cura a Carattere Scientifico (IRCCS), Rozzano, MI, Italy; 6 Medical Oncology Unit, Istituto Clinico Mater Domini Humanitas, Castellanza, Varese, Italy

**Keywords:** breast cancer, Li-Fraumeni Syndrome, low variant allele frequency, post-zygotic mosaicism, *TP53*

## Abstract

Li-Fraumeni syndrome (LFS) is a rare hereditary cancer predisposition syndrome caused by pathogenic variants in the *TP53* gene. The increasing use of next-generation sequencing (NGS) in germline testing has led to more frequent detection of *TP53* variants with low variant allele frequency (VAF), raising diagnostic challenges in distinguishing true germline alterations from post-zygotic mosaicism or clonal hematopoiesis and circulating tumor DNA, with relevant clinical and counseling implications. We report a 30-year-old woman diagnosed with early-onset HER2-positive breast cancer who underwent genetic counseling for suspected hereditary cancer predisposition. Germline NGS identified a pathogenic *TP53* variant with low VAF (16.7%) in peripheral blood, suggestive of a non-germline event. To clarify its origin, additional molecular analyses were performed on non-hematopoietic tissues. The same variant was detected in buccal cells (ectoderm) and skin fibroblasts (mesoderm), and subsequently confirmed in normal colonic mucosa (endoderm), demonstrating the presence of the pathogenic variant across all three embryonic germ layers. These findings support an early post-zygotic origin and confirm generalized post-zygotic mosaicism. Based on this molecular characterization, a personalized surveillance and management strategy was implemented. This case provides rare and robust molecular evidence of *TP53* post-zygotic mosaicism involving all three embryonic germ layers in a patient with early-onset breast cancer. It highlights the importance of extended tissue testing to correctly interpret low-VAF *TP53* variants and to distinguish mosaicism from clonal hematopoiesis and circulating tumor DNA. Recognition of *TP53* mosaicism has significant implications for cancer risk assessment, surveillance strategies, and genetic counseling, underscoring the need for individualized clinical management in the absence of specific guidelines.

**Scope Statement:**

This case report highlights the increasing diagnostic challenges posed by low-VAF *TP53* variants detected through germline NGS and emphasizes the critical importance of extended tissue testing to distinguish true germline variants from post-zygotic mosaicism and clonal hematopoiesis or circulating tumor DNA. Our findings have direct clinical implications for cancer risk assessment, surveillance strategies, and genetic counseling, particularly in young patients with early-onset tumors belonging to the Li-Fraumeni syndrome spectrum. To our knowledge, this represents a rare and well-documented example of *TP53* post-zygotic mosaicism involving all three embryonic germ layers, supported by comprehensive molecular characterization. We believe that this report provides valuable insights for clinicians, geneticists, and oncologists involved in hereditary cancer risk assessment and management.

## Introduction: Li-Fraumeni syndrome overview

Li-Fraumeni syndrome (LFS), also referred to as *TP53* -related cancer syndrome, is a rare hereditary cancer predisposition syndrome caused by pathogenic variants (PVs) in the *TP53* gene (1–3). It is characterized by a markedly increased lifetime risk of cancer, with a broad tumor spectrum that includes breast cancer, soft tissue and bone sarcomas, central nervous system tumors, and adrenocortical carcinoma (1–4). Cancer onset is often early, with approximately half of affected individuals developing malignancies before the age of 40, and survivors are at increased risk for multiple primary tumors (4–6).

Among LFS-associated malignancies, breast cancer represents the most frequent tumor in women and often occurs at a young age, frequently before menopause. These tumors are commonly high grade, hormone receptor–positive, and HER2-amplified, and are associated with an increased risk of bilateral disease and second primary cancers (5–7). Early-onset HER2-positive breast cancer has therefore been recognized as a clinical red flag for *TP53* testing (8).

LFS is inherited in an autosomal dominant manner with high but variable penetrance. While most affected individuals carry heterozygous germline *TP53* PVs, approximately 7–20% of cases arise from *de novo* mutations (6, 9). Historically, diagnosis relied on family history–based criteria, including the classic Li-Fraumeni and modified Chompret criteria (1, 10). However, the increasing availability of genetic testing has revealed substantial phenotypic heterogeneity, suggesting that cancer risk may be influenced not only by variant type but also by modifying factors and the developmental timing of the mutational event (6, 11–13).

The *TP53* gene encodes the p53 tumor suppressor protein, a key regulator of genomic stability, cell cycle arrest, apoptosis, senescence, and DNA repair (14, 15). More than 300 germline *TP53* variants have been reported, most frequently missense mutations affecting the DNA-binding domain (12, 16). While true germline *TP53* PVs are estimated to occur in approximately 1 in 3,000–10,000 individuals, population-based sequencing studies suggest a higher prevalence of potentially pathogenic variants, many of which may not confer the classical LFS phenotype (12, 16).

The widespread adoption of next-generation sequencing (NGS) in hereditary cancer testing has introduced new interpretative challenges. In particular, *TP53* PVs detected at low variant allele frequency (VAF), typically below 30%, complicate the distinction between true germline variants, post-zygotic mosaicism, and clonal hematopoiesis or ctDNA (17–20). This distinction is clinically critical, as each scenario carries different implications for cancer risk assessment, surveillance strategies, family counseling, and reproductive decision-making (18, 19, 21).

Post-zygotic *TP53* mosaicism results from post-zygotic mutational events and may involve one or multiple tissues, depending on the developmental timing of the mutation (18, 19, 22). Early embryonic events may result in widespread mosaicism affecting tissues derived from different embryonic germ layers, whereas later events are often restricted to specific cell lineages. In contrast, clonal hematopoiesis reflects the expansion of a mutant hematopoietic clone and does not necessarily imply increased cancer risk in non-hematopoietic tissues (21).

In this context, analysis of additional tissues beyond peripheral blood—including ectodermal, mesodermal, and endodermal derivatives—has emerged as a valuable strategy to clarify the biological origin of low-VAF *TP53* variants and to guide clinical management (18, 19, 22).

Here, we report the case of a young woman with early-onset HER2-positive breast cancer in whom a pathogenic *TP53* variant was detected at low VAF in blood. Through a multi-tissue molecular approach, we demonstrate generalized post-zygotic *TP53* mosaicism involving all three embryonic germ layers, highlighting the diagnostic value and clinical relevance of extended tissue testing in the interpretation of *TP53* variants detected by NGS.

## Case report: a young woman with breast cancer

This case concerns a female patient in her third decade of life who presented with a palpable mass in the right breast in early 2020. Concerned, she underwent a core biopsy, which confirmed an invasive carcinoma of no special type, Grade 3. The tumor expressed high levels of estrogen (ER 90%) and progesterone (PgR 50%) receptors, with a Ki-67 proliferation index of 30%, and was strongly positive for HER2 (3+), indicating an aggressive subtype.

To ensure the patient was eligible for treatment, a complete staging workup was performed. This included a contrast-enhanced total-body CT with bone window settings, as well as dedicated CT scans of the brain, chest, and abdomen. A total-body bone scintigraphy was also conducted. All diagnostic imaging confirmed the absence of secondary lesions or metastatic disease.

**Figure 1:**
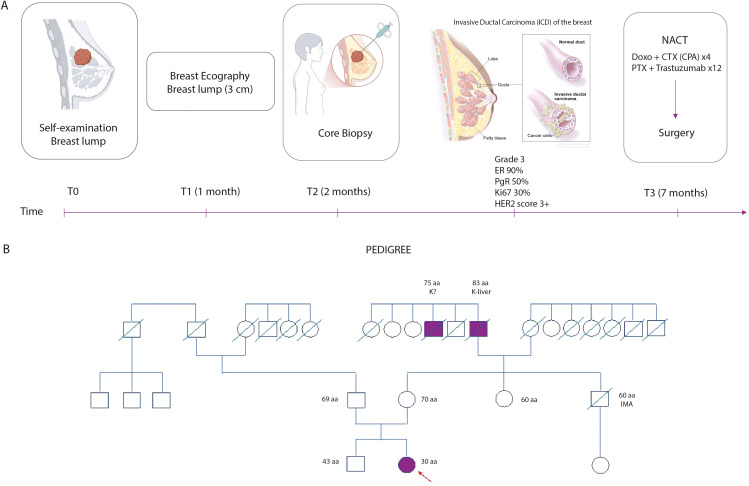
**(A)** Clinical timeline from self-detection of a breast lump to diagnosis and treatment. Diagnostic work-up included breast ultrasound, core biopsy confirming invasive ductal carcinoma (IDC), and neoadjuvant chemotherapy (NACT) followed by surgery. Tumor characteristics are reported. **(B)** Family pedigree; the proband is indicated by the arrow. Filled symbols denote affected individuals, slashed symbols indicate deceased individuals, and ages and cancer diagnoses are reported where available.

She initiated neoadjuvant therapy at 6 months, consisting of four cycles of Adriamycin and Cyclophosphamide, followed by weekly Paclitaxel and 12 administrations of Trastuzumab. Prior to chemotherapy, she underwent oocyte cryopreservation for fertility preservation and has been receiving LH-RH analog therapy (**Figure 1A**).

Her medical history included a diagnosis of polycystic ovary syndrome in 2019. She followed a Mediterranean diet, did not engage in regular physical activity, and quit smoking at the beginning of cancer treatment after previously smoking 2–4 cigarettes per day. Menarche occurred at age 14, with regular menstrual cycles, and she has never been pregnant.

A three-generation family history revealed no significant oncological findings on her paternal side, although the family was small. On her maternal side, her grandfather died of liver cancer at 83, and one of his brothers died at 75 from an unspecified cancer. There were no cases of breast or ovarian cancer in her immediate family (**Figure 1B**) which support a Hereditary Breast and Ovarian Cancer (HBOC) syndrome.

Given her early age at diagnosis, the patient was referred for genetic counseling to evaluate the possibility of an inherited cancer predisposition. Standard pre-test counseling was provided, including information on sporadic, familial, and hereditary cancers, as well as the implications of germline testing. Initial testing of *BRCA1* and *BRCA2* was recommended, and a broader 14-gene panel—including high-risk genes such as *TP53*—was proposed given her early-onset disease.

She initially consented to *BRCA1* and *BRCA2* testing and deferred panel genes analysis. Post-test counseling revealed no pathogenic variants in *BRCA1/2*. Multigene panel testing was subsequently discussed, including clinical implications, and limitations, such as variants of uncertain significance, mosaicism, or splice-site variants. The patient then provided informed consent for the 14-gene panel, and a peripheral blood sample was collected for analysis.

Then, during follow-up post-test counseling, the results of the *TP53* genetic analysis were delivered and explained to her.

## Molecular genetic analysis

### *TP53* mutational analysis

**Figure 2:**
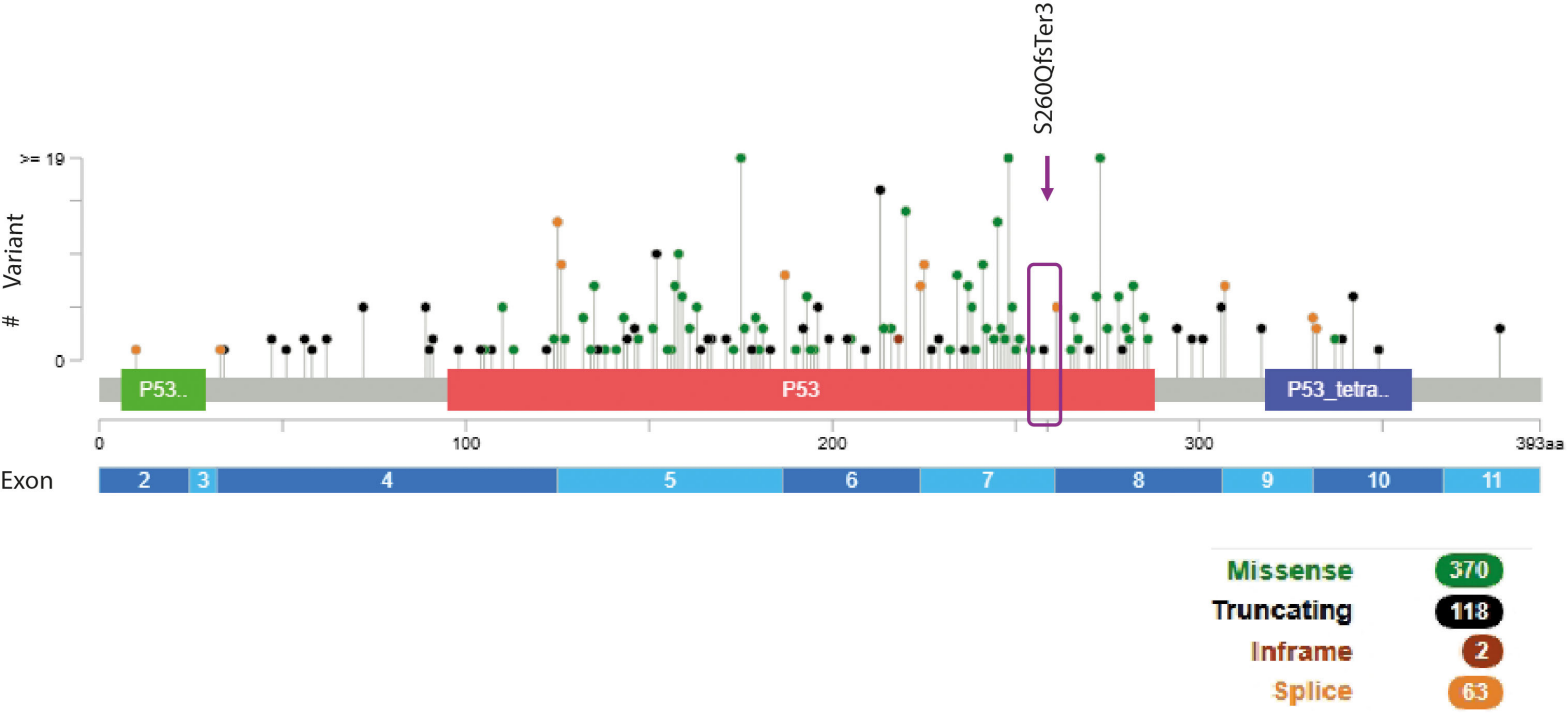
Lollipop diagram showing known pathogenic and likely pathogenic *TP53* variants compiled from the Leiden Open Variation Database, ClinVar, and cBioPortal. Each vertical line represents a single genetic variant, including single-nucleotide substitutions and small indels. Labels highlight the amino-acid changes at selected nucleotide position of the identified *TP53* pathogenic variant. Large deletions and duplications/amplifications are not included. Violet box indicates the locus of patient’s variant. Figure adapted from images created with cBioPortal, last access March 25, 2026.

**Figure 3:**
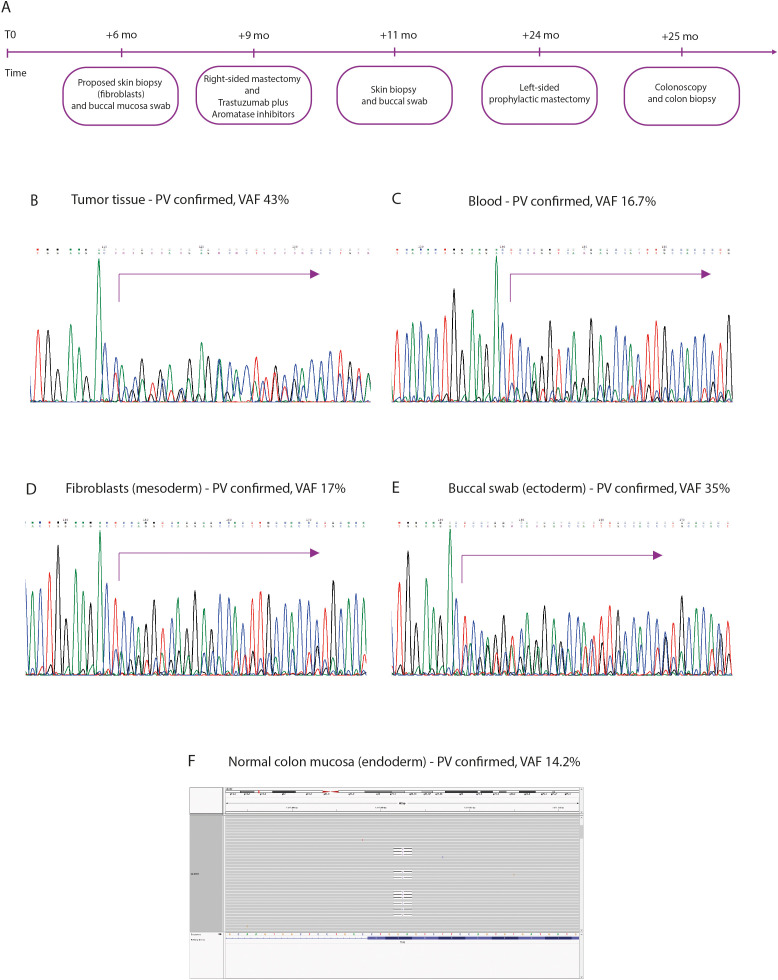
PV, pathogenic variant; VAF, variant allele frequency. **(A)** Clinical timeline and sampling procedures. **(B)** Electropherogram of the pathogenic variant, c.778_779delTC, p. (Ser260GlnfsTer3), in exon 7 of *TP53* gene in tumor tissue. **(C)** Electropherogram of the same pathogenic variant, in blood sample. **(D–F)** Demonstrate confirmation of the *TP53* pathogenic variant in tissues derived from all three embryonic germ layers: fibroblasts (mesoderm), buccal swab (ectoderm), and normal colonic mucosa (endoderm) – BAM visualization, with variable VAFs consistent with post-zygotic mosaicism. Electropherogram by Chromas 2.6.6.

Next-generation sequencing (NGS), confirmed by Sanger sequencing, identified a pathogenic *TP53* variant, NM_000546.6: c.778_779delTC, p.(Ser260GlnfsTer3), in exon 7 (**Figure 2**) with an allelic frequency (VAF) of 43% in tumor tissue derived from ultrasound-guided core needle biopsy of the nodular lesion described in the lower-inner quadrant of the right breast (**Figure 3B**) and of 16.7% in blood (**Figure 3C**). This VAF is below the 30% threshold, suggesting the variant is not germline and does not support a diagnosis of LFS, which typically requires a germline pathogenic *TP53* mutation with a VAF of 30–70% (18) with a suggestive phenotype.

A VAF below 30% usually indicates a somatic variant, potentially arising from post-zygotic mosaicism or clonal hematopoiesis (17) and circulating tumor DNA (ctDNA) (20). Distinguishing between these possibilities is clinically important:

- Detection of the variant in other tissues (e.g., skin fibroblasts, oral mucosa) supports post-zygotic mosaicism.
- Absence in non-tumor tissues favors clonal hematopoiesis or ctDNA, although mosaicism cannot be entirely excluded.

The patient therefore underwent further testing with skin biopsy and oral mucosal swab.

### *TP53* analysis in buccal swab (sanger sequencing)

The heterozygous variant was detected in ectodermal DNA from the buccal swab and confirmed in a second independent sample. The buccal swab may be contaminated by leukocytes from saliva. However, the estimated allelic fraction-higher than that observed in blood-strongly suggests the presence of the variant in buccal epithelial cells derived from the ectoderm. The VAF in buccal DNA was approximately 35%, supporting the presence of the variant outside the hematopoietic lineage (**Figure 3B**).

### *TP53* analysis in skin fibroblasts (sanger sequencing)

The variant was also detected in mesodermal DNA from cultured fibroblasts (skin biopsy) with a VAF of approximately 17%, confirmed in a second sample (**Figure 3C**). The use of cultured fibroblasts, rather than a direct skin biopsy, was essential to exclude potential DNA contamination from circulating blood cells, thereby ensuring that the detected variant was truly representative of the mesodermal lineage. The presence of the same variant in tissues derived from different embryonic layers (ectoderm and mesoderm) supports the hypothesis of post-zygotic mosaicism, likely originating from an early embryonic event before gastrulation.

Clonal hematopoiesis and ctDNA were excluded, prompting further investigation in endoderm-derived tissue to better assess cancer risk.

### *TP53* analysis in colon biopsies

A colon biopsy was proposed, and in August 2022, the patient underwent colonoscopy with sampling of normal colon and rectal tissue.

The heterozygous *TP53* variant was detected by NGS analysis in normal colon tissue with a VAF of approximately 14.2% (**Figure 3D**), further supporting post-zygotic mosaicism hypothesis. Its presence was thus confirmed across tissues derived from embryonic layers suggests the variant arose early in embryogenesis.

Following the delivery of the genetic results from the colonic biopsies, whose implications were explained during post-test genetic counseling, and after a discussion concerning the current state of knowledge as well as the feasible and non-feasible preventive strategies for the various organs, and in the absence of established and available guidelines on the matter, the patient was referred to a reference center for Li-Fraumeni syndrome.

The patient’s cancer treatment journey began with a right mastectomy at the end of NACT which revealed ypTisN0 on histological examination. Postoperatively, she received trastuzumab therapy. One year later, she underwent a prophylactic left mastectomy. She is currently receiving treatment with exemestane in combination with an LH-RH analog and remains under regular oncological surveillance.

## Discussion

### *TP53* mosaicism as a diagnostic and clinical challenge

The widespread use of NGS in germline testing has significantly increased the detection of *TP53* pathogenic variants with low variant allele frequency (VAF) (17, 18). While such findings may occasionally reflect true germline variants diluted by technical (i.e., DNA quality and specimen type, PCR artifacts, mapping errors, read depth) or biological factors (i.e., Copy Number Alterations – CNA, ctDNA), they more frequently raise the differential diagnosis between post-zygotic mosaicism, clonal hematopoiesis, and, less commonly, tumor DNA contamination (18, 19, 21, 23). Correct interpretation is essential, as each scenario carries profoundly different implications for cancer risk assessment, surveillance strategies, and genetic counseling.

In the context of *TP53*, in accordance with current clinical guidelines, a VAF below 30% in peripheral blood should prompt careful evaluation rather than immediate classification as classical Li-Fraumeni syndrome (LFS) (18, 24). Clonal hematopoiesis represents a frequent confounder in adults, increasing with age, and in metastatic diseases, but post-zygotic *TP53* mosaicism must also be considered, particularly in patients presenting with early-onset tumors belonging to the LFS spectrum, such as HER2-positive breast cancer diagnosed at a young age (8, 18, 21).

### When to suspect *TP53* mosaicism

This case highlights several elements that should raise suspicion for *TP53* mosaicism: detection of a clearly pathogenic *TP53* variant with low VAF in blood-derived DNA, the presence of an early-onset “red flag” tumor, and the absence of a strong family history consistent with autosomal dominant inheritance (6, 8, 10, 18). In such scenarios, reliance on blood-based testing alone may lead to misclassification and inappropriate management, either through overestimation or underestimation of cancer risk.

Approximately 7–20% of individuals with *TP53* germline PVs carry *de novo* mutations (6, 9, 11, 25), further complicating interpretation when parental testing is uninformative or unavailable. These observations reinforce the need for a comprehensive diagnostic approach integrating molecular findings with clinical context.

### Role of multi-tissue testing and embryonic origin

Extending molecular analysis to additional non-hematopoietic tissues represents a key strategy to distinguish post-zygotic mosaicism from clonal hematopoiesis (18, 19, 22). Testing tissues derived from different embryonic germ layers provides insight into the developmental timing of the mutational event. For instance, detection of the same *TP53* PV in ectodermal, mesodermal, and endodermal tissues, as observed in this patient, strongly supports an early post-zygotic origin occurring before gastrulation, resulting in generalized post-zygotic mosaicism (18, 19, 22).

This distinction is clinically meaningful. Although post-zygotic *TP53* mosaicism differs in its biological origin from constitutional heterozygous variants, these individuals should be considered as true LFS patients. Widespread mosaicism involving multiple tissues may confer a cancer risk that approaches that of classical LFS, particularly with respect to sensitivity to ionizing radiation and the potential development of multiple primary tumors (18, 24, 26). However, while multi-tissue testing remains the diagnostic gold standard for confirming extensive mosaicism, it may be more practical in routine clinical settings to prioritize less invasive samples, such as hair follicles or saliva, to avoid the challenges associated with skin or colonic biopsies (27, 28). Extensive sampling of multiple germ layers should, therefore, be considered a valuable illustrative approach for complex cases or when significant diagnostic ambiguity persists, rather than a systematic requirement for all patients.

### Clinical management implications

At present, no specific guidelines address cancer surveillance and management in individuals with *TP53* post-zygotic mosaicism. In the absence of evidence-based recommendations, management must be individualized, adopting surveillance strategies similar to those recommended for LFS appears reasonable, particularly regarding minimization of radiation exposure and preference for MRI-based imaging modalities (24, 26, 29, 30). Furthermore, genetic counselling and management should be identical to that of patients carrying *de novo* heterozygous variants, including the offer of presymptomatic testing for their offspring. Referral to specialized centers with expertise in *TP53* -related cancer syndromes is strongly recommended to support multidisciplinary decision-making and long-term follow-up.

### Family counseling and reproductive considerations

*TP53* mosaicism also raises important issues regarding familial risk and reproductive counseling. While germline involvement cannot be assumed, it cannot be definitively excluded, particularly in cases of early embryonic mosaicism affecting multiple tissues (18, 19). Consequently, predictive testing in offspring should be proposed, and reproductive options such as prenatal or preimplantation genetic testing may be considered when a pathogenic variant has been identified (5, 9, 30, 31). These discussions require careful genetic counseling to address uncertainty, psychological impact, and ethical considerations.

## Conclusions

The detection of *TP53* pathogenic variants with low variant allele frequency is becoming increasingly common with the expanding use of next-generation sequencing in hereditary cancer testing (17, 18). This case underscores the importance of moving beyond blood-based analysis when interpreting such findings and demonstrates the value of multi-tissue testing to distinguish post-zygotic mosaicism from clonal hematopoiesis (18, 19, 21).

Demonstration of a *TP53* pathogenic variant across all three embryonic germ layers provides strong evidence of early post-zygotic mosaicism and has direct implications for cancer risk assessment, surveillance strategies, and clinical management (18, 19, 22). In the absence of dedicated guidelines, a pragmatic and individualized approach—integrating molecular data, tumor characteristics, and clinical context—is essential.

Accurate recognition and classification of *TP53* mosaicism are critical to avoid misdiagnosis, optimize surveillance, and deliver appropriate genetic counseling. As genomic testing continues to evolve, increased awareness and systematic evaluation of mosaic states will play a key role in refining personalized care for individuals with *TP53* -related cancer predisposition syndromes.

## Data Availability

This study is a case report, and no datasets were generated or deposited in public repositories. The data supporting the findings are contained within the article and are derived from the patient's clinical records. Due to ethical and privacy considerations, these data are not publicly available. De-identified data may be made available from the corresponding author upon reasonable request and subject to institutional approval.

## Appendix

### Materials and methods

Patient Samples

Written informed consent was obtained for germline genetic analysis using blood, buccal swabs, fibroblasts, and tumor tissue, including NGS panel testing and Sanger sequencing.

DNA and RNA Extraction

- DNA from blood, buccal swabs, and fibroblasts was extracted using QIAamp DNA Mini Kit (Qiagen).
- For FFPE tissue, 8 µm sections were macrodissected from H&E-stained slides, and DNA was extracted using QIAamp DNA FFPE Tissue Kit (Qiagen).

Next-Generation Sequencing (NGS)

- NGS was performed using the Illumina *SOPHiA DDM™ CUSTOM Bundle Solution* (SOPHiA GENETICS™), kit *Custom Hereditary Cancer Solution* (SOPHiA GENETICS™), covering 70 high-risk cancer genes.
- Fourteen genes associated with high breast/ovarian cancer risk (*ATM*, *BRCA1*/*2*, *BRIP1*, *CDH1*, *CHEK2*, *NBN*, *NFI*, *PALB2*, *PTEN*, *RAD51C/D*, *STK11*, *TP53*) were prioritized per NCCN guidelines.
- Libraries were prepared from 50 ng genomic DNA, enriched for target regions, and sequenced Platform *MiSeq Illumina DX* with paired-end 150-bp reads.
- NGS analysis utilized a validated panel with detection thresholds of 20% for SNPs and 15% for small indels, ensuring robust identification of low-level mosaicism. To guarantee precise VAF estimation, a mean coverage depth of 800x was maintained across all targeted exons.

Data Analysis and Variant Interpretation

- FASTQ files were analyzed with the *Sophia Genetics DDM™* platform.
- Variants were called against hg19, filtered for quality, and classified according to ACMG, ENIGMA, IARC *TP53* database, and Global Variome shared LOVD as pathogenic, likely pathogenic, VUS, likely benign, or benign.

Sanger Sequencing

- *TP53* exon 7 mutations were validated via PCR (AmpliTaq Gold 360 Master Mix) followed by BigDye terminator sequencing on an ABI 3130.
- Sequences were analyzed using Sequencing Analysis (Chromas 2.6.6) and Variant Reporter software against hg19/GRCh37.
